# Neuron and Brain Maturation 2.0

**DOI:** 10.3390/ijms242317113

**Published:** 2023-12-04

**Authors:** Luca Bonfanti, Sébastien Couillard-Després

**Affiliations:** 1 Neuroscience Institute Cavalieri Ottolenghi (NICO), 10043 Orbassano, Italy; 2 Department of Veterinary Sciences, University of Turin, 10095 Torino, Italy; 3 Spinal Cord Injury and Tissue Regeneration Center Salzburg (SCI-TReCS), Paracelsus Medical University, 5020 Salzburg, Austria; 4 Institute of Experimental Neuroregeneration, Paracelsus Medical University, 5020 Salzburg, Austria; 5 Austrian Cluster for Tissue Regeneration, 1200 Vienna, Austria

The mammalian central nervous system (CNS) is built up during embryogenesis by neural stem cells located in the periventricular germinal layers which undergo multiple division cycles. The newly born neuroblasts subsequently migrate away to reach their final integration targets. The whole process is designated as neurogenesis [1,2,3]. At the time of birth, the mammalian brain and spinal cord are largely assembled. Yet, a great variety of ongoing plastic processes are still required to complete the neural circuit assembly and refinement, and those processes are strongly modulated by external environmental cues [4]. Hence, the mature nervous system will be shaped through postnatal and young ages, allowing experiences to impress on brain maturation and to lead to an individualized “vision of the world”, which persists throughout life [5].

A substantial stabilization of the adult neural circuits will be obtained via progressive maturation, at the single cell scale as well as the regional scale. The maturation processes proceed with remarkably heterogenous timing, and structural plasticity is maintained at specific locations to a certain degree for life [2,4]. Hence, after the end of developmental events, which shape embryonic neurogenesis, the residual tailoring capacity that remains active mostly represents a juvenile tool that “built up the vision of the world” [5,6,7]. However, many neurobiologists consider postnatal and adult cellular plasticity primarily as a reservoir for brain repair [8]. The latter perspective has been celebrated after the discovery of neural stem cells and adult neurogenesis. Yet, the huge number of studies published since then indicate that brain regeneration is currently beyond the bounds of possibility in mammals [9]. Although hopes for brain repair through the stimulation of endogenous structural plasticity or via the transplantation of various types of stem cells still remain, so far, consistent therapeutic results in humans have only been reached through common rehabilitation techniques targeting the compensatory mechanisms of synaptic plasticity. On the other hand, increasing evidence demonstrates that neurological and/or psychiatric disorders emerge when the correct final assembly and maturation of the neural circuits are perturbed [10,11].

Across the years, the emphasis placed on the enablement of brain repair through brain structural plasticity (e.g., neural stem cell biology, adult neurogenesis) overshadowed the importance of postnatal brain maturation. Both processes are closely related since the maintenance of neuronal “immaturity” is a prerequisite for structural plasticity to occur. Processes of neuronal maturation, namely well-orchestrated sequences of molecular and cellular modification, ranging from stem cell determination to the integration of functional neurons, govern structural plasticity during the prenatal as well as postnatal CNS development (Figure 1) [12]. Recent research revealed that certain neural cell markers characterizing maturational phases (e.g., nestin, doublecortin, PSA-NCAM, NeuN) are shared through widely different processes, such as embryonic neurogenesis, adult neurogenesis, and dormant immature neurons (Figure 1) [4,13].

The promiscuity of available markers adds to the complication of understanding brain plasticity and maturation and calls into question previously accepted dogmas. For instance, the detection of prenatally generated, non-newly generated “immature” neurons, based on their expression of markers of neuronal immaturity in adult cortical regions known to be devoid of active stem cells or neurogenesis [13]. In some brain regions, these immature neurons continue to exist throughout a lifetime without functionally connecting with their siblings, which contradicts the dogma on the necessity for immature neurons to establish synaptic connections with their target in order to survive. Most astonishingly, neurons that remained immature, in some instances, for decades can progressively “awaken” as fully mature neurons and functionally integrate into pre-existing neuronal circuits, in a sort of “neurogenesis without division” [14]. Such heterogeneity in brain structural plasticity, involving canonical and non-canonical neurogenic processes, sharing some of the cell markers, compounds the challenge of its understanding.

Unexpectedly, significant interspecies variations in mammals have recently come to light regarding the coexisting types of postnatal brain structural plasticity [4], foremost in respect to maturation processes. Comparative neuroplasticity studies have unveiled a relative decrease in stem cell-driven adult neurogenesis in large-brained, long-living mammals as compared to rodents. Simultaneously, an opposite trend emerges with the occurrence and quantity of immature, “dormant” neurons [4,15,16]. Accumulating data substantiate the hypothesis that neuronal maturation in mammals with extended lifespans, such as humans, unfolds over an extended duration [4]. Consequently, the outcome of this protracted maturation is the enduring presence of a reservoir of immature neurons, furnishing the aging brain with a supply of “young cells”. For instance, the maturation of hippocampal granule cells in the adult neurogenesis of sheep and primates far exceeds the 3–4 weeks occurring in rodents, reaching 3 months [17] and 6 months [18], respectively. These observations suggest that a sort of “dilatation” in the period of plasticity could be associated with increasing lifespans. Even for synaptic plasticity, a process that is well-conserved among mammals (i.e., low interspecies variation in synaptic density and structure [19,20]), postsynaptic density maturation has been shown to progressively slow down with age in humans in comparison to rodents [21].

Furthermore, focusing on the most striking example of the endogenous modulation of neuronal maturation, namely the so-called cortical “immature” or “dormant” neurons [14], a recent study carried out using a DCXCreERT2/Flox-EGFP transgenic mouse model showed that a small population of these dormant cells continue to wake and mature even at advanced ages (at least 15 months). However, the timespan required for the maturation processes to complete increases as the age progresses [14]. Of interest, another recent report carried out on postmortem-fixed human brains of individuals from neonatal to very old stages [22] demonstrated a long-lasting persistence of numerous cortical “immature” neurons in the whole cerebral cortex.

Taken together, all these data spanning different species and ages indicate that slowing down neuronal maturation to extend the period of immaturity might have been a useful strategy for accommodating the brain with “young” cells and neotenic features over longer windows of time, even in the absence of active neural stem cells. Long-living, large-brained mammals seem to have explicitly favored this strategy over the stem cell-based neurogenesis, an element of uttermost importance for the translation of interventions targeting brain plasticity. Therefore, there is an urgent need for a more comprehensive understanding of neuronal and brain maturation from several angles, from the cellular/molecular scale [23,24,25] to the complexity of the whole brain. Likewise, the remarkable interspecies differences and their evolutionary implications [4,26,27] must be further deciphered. Finally, new or better technologies will be required to enable non-invasive, functional imaging and shed light on the complex dynamic of maturation processes in humans [11].

## Figures and Tables

**Figure 1:**
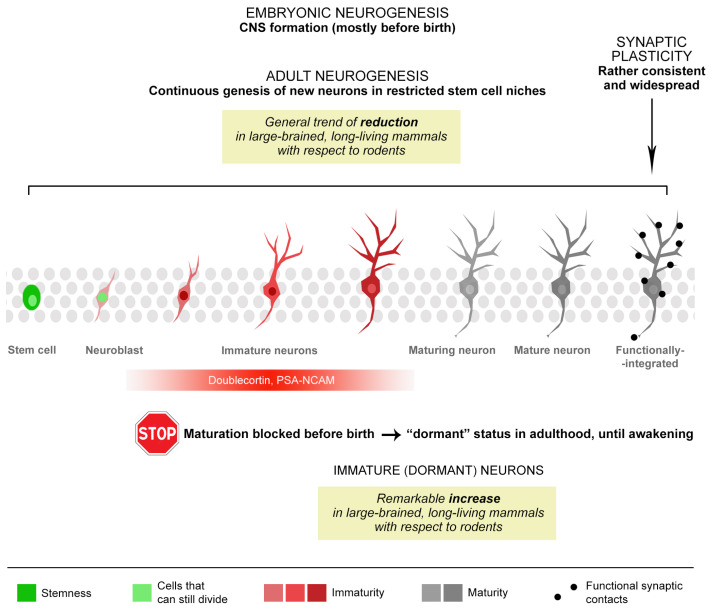
Different levels of heterogeneity in the types and occurrence of brain structural plasticity, some of which are linked to neuronal maturation. At least three types of structural modifications can be found, spanning from embryogenesis to adulthood: synaptic plasticity (acting on pre-existing neurons) and three different types of neurogenic processes (embryonic neurogenesis, adult neurogenesis, and “immature” or “dormant” neurons). After CNS formation (mostly occurring before and around birth) new neurons can be structurally and functionally integrated into the neural circuits, both with and without the persistence of stem cells/cell division (adult neurogenesis and immature neurons, respectively). Further heterogeneity arises from remarkable interspecies differences that exist among mammals and regarding the occurrence/location/rate of different neurogenic processes, linked to brain size and lifespan.
